# The impact of time length to Boolean remission for tight disease activity control after acquisition in rheumatoid arthritis patients

**DOI:** 10.1038/s41598-023-39711-4

**Published:** 2023-08-25

**Authors:** Ichiro Yoshii, Tatsumi Chijiwa, Naoya Sawada

**Affiliations:** 1 Department of Rheumatology and Musculoskeletal Medicine, Yoshii Hospital, 6-7-5 Nakamura-Ohashidori, Shimanto, Kochi 787-0033 Japan; 2 Department of Rheumatology, Kochi Memorial Hospital, 4-13 Shiromi-Cho, Kochi, Kochi 780-0824 Japan; 3 Department of Rheumatology, Dohgo Onsen Hospital, 21-21 Himetsuka-Otsu, Matsuyama, Ehime 790-0858 Japan

**Keywords:** Rheumatoid arthritis, Prognostic markers, Rheumatology, Rheumatic diseases

## Abstract

Clinical importance of time length from initiation under treat-to-target (T2T) strategy to acquisition of clinical remission (TL) in treating patients with rheumatoid arthritis (RA) on disease activity control, daily activities, and quality of life maintenance was investigated. In patients who achieved Boolean remission once or more, relationship between TL and patients’ background data at initiation, and relationship between TL and mean simplified disease activity score (SDAI), Health Assessment Questionnaire Disability Index (HAQ-DI) score, pain score with visual analog scale (PS-VAS), Sharp/van der Heijde Score (SHS) and quality of life score (QOLS) at the first remission and thereafter were evaluated statistically. Patients were divided into two groups whether TL was within 6 months or longer (G ≤ 6 and G > 6). Change of the parameters and Boolean remission rate (BRR) after the first remission between the two groups were compared statistically. In 465 patients, TL correlated significantly with the SDAI score, the HAQ score, PS-VAS, SHS, and the QOLS after the remission. The SDAI score and the BRR after the remission were significantly better in the G ≤ 6 than in the G > 6. TL is an important key to guarantee good and stable clinical course in treating under T2T.

## Introduction

There is a broad consensus that clinical remission should be the initial goal for treating rheumatoid arthritis (RA)^1–5^, because the majority of clinical practices and trials reported the benefit of attaining clinical remission on radiographic damage disturbance and daily activity maintenance^6–14^. Clinical remission is indexed with Boolean criteria, simplified disease activity index (SDAI) score^10, 15, 16^, clinical disease activity index (CDAI) score^16^, and 28-joint disease activity score using C-reactive protein (DAS28-CRP)^17^. In clinical practice, sustaining clinical remission is the treatment goal for patients with RA that would improve the radiographic destruction of joints, daily activities in life (ADL), and quality of life (QOL)^18–23^. For these patients, Boolean remission criteria may be the most stringent criteria, and it would guarantee better clinical outcomes for both disease activity and radiographic progression^21, 24–26^.

In contrast, despite the strong recommendation by the European League Against Rheumatism (EULAR) that clinical remission in RA should be achieved within 3–6 months from the first visit to a rheumatologist^3–5^, the impact of early achievement of clinical remission on clinical outcomes is not discussed enough. Although  in a literature, it is suggested that the early acquisition of clinical remission can achieve better clinical outcomes as a result of tight disease control^27^, it was reported before advocating the treat-to-target (T2T) strategy. To the best of our knowledge, no studies have reported the impact of early achievement of clinical remission under the T2T strategy on comprehensive clinical courses.

Hence, we investigated this issue using small cohort data; the impact of time length to achieve Boolean remission on clinical outcome was evaluated statistically and discussed why 3–6 months to achieve is an appropriate target.

## Results

### Parameters and regression analysis at baseline

A total of 685 patients with RA were recruited. Of these, 465 patients had achieved Boolean remission once or more. Out of 465 patients, females comprised 343 (73.7%), and the mean age was 67.8 years (ranging from 21 to 95 years). These patients were analyzed in the study. The mean disease duration at the first visit was 6.1 years (range from 1 month to 45 years), and there was no case that demonstrated Boolean remission at the first visit. The mean anti-cyclic citrullinated peptide antibodies (ACPA) titer was 197.4 U/L, and 336 (72.3%) patients were positive for ACPA, whereas the mean RF titer was 95.2 IU/mL, and 350 (75.3%) patients were positive for RF. The mean follow-up length was 71.5 months (range 36–122 months; median 85 months) and the mean time length from the first visit to the first Boolean remission was 8.1 months (range 1–111 months; median 4 months). The mean SDAI score, Health Assessment Questionnaire Disability Index (HAQ-DI) score, pain score using a visual analog scale (PS-VAS), Sharp/van der Heijde score (SHS), and the quality of life score (QOLS) at the first visit were shown in Table 1.

**Table 1:** Demographic and clinical characteristics of the patient and each group at baseline and time length of each period.

	In all	G ≤ 6	G > 6	*p*-value
Cases	465	323	142	
Female (%)	343 (73.7%)	245 (76.8%)	98 (69.0%)	0.12
Age	67.8 (13.9), 69, 21–95	67.6 (14.1), 69, 25–95	68.2, 13.4, 69, 21–93	0.81
Disease duration	6.1 (7.9), 3.75, 0.1–45	**5.4, 7.5, 3, 0.1–45**	**7.7 (8.6), 5, 0.1–45**	**< 0.001**
ACPA positive (%)	336 (72.3%)	232 (71.8%)	104 (73.2%)	0.75
RF positive (%)	350 (75.3%)	243 (75.2%)	107 (75.4%)	0.98
MTX use	254 (54.6%)	180 (55.7%)	74 (52.1%)	0.54
b/tsDMARD use	44 (9.5%)	23 (7.1%)	21 (14.8%)	0.18
GCS use	128 (27.5%)	81 (25.1%)	47 (33.1%)	0.21
TJC	2.3 (3.0), 1, 0–20	**2.2 (3.1), 1, 0–20**	**2.5 (2.9), 2, 0–19**	**< 0.05**
SJC	3.9 (4.8), 2, 0–28	4.0 (5.2), 2, 0–28	3.8 (4.8), 3, 0–16	0.24
PGA	2.9 (2.8), 2, 0–10	**2.8 (2.9), 2, 0–10**	**3.3 (2.7), 3, 0–10**	**< 0.05**
EGA	2.2 (2.1), 2, 0–10	2.1 (2.1), 2, 0–10	2.3 (1.9), 2, 0–10	0.16
CRP	14.0 (27.0), 2.7, 0–207.0	15.0 (29.0), 2.8, 0–207.0	14.0 (22.0), 2.7, 0–144.0	0.25
DAS28	3.9 (1.2), 2.5, 2.6–6.4	3.8 (1.2), 2.4, 2.6–6.4	3.9 (1.0), 2.6, 2.6–5.5	0.13
CDAI	11.8 (10.5), 8.5, 4–66	11.8 (11.3), 7.5, 7–66	11.9 (8.6), 10.8, 4–56	0.45
SDAI	13.3 (12.2), 8.8, 4.3–71.1	13.3 (12.9), 7.7, 7.4–71.1	13.3 (9.8), 11.3, 4.3–64.4	0.68
HAQ-DI	0.467 (0.582), 0.250, 0–2.75	**0.418 (0.564), 0.125, 0–2.75**	**0.578 (0.607), 0.375, 0–2.625**	**< 0.01**
PS-VAS	33.2 (29.5), 25, 0–100	**30.3 (28.9), 20, 0–100**	**40.5 (29.6), 47.5, 0–100**	**< 0.001**
SHS	47.9 (62.9), 22, 0–340	**28.3 (51.3), 19, 0–296**	**69.1 (78.9), 35, 0–340**	**< 0.001**
QOLS	0.83 (0.12), 0.89, 0.19–0.94	**0.85 (0.13), 0.89, 0.19–0.94**	**0.81 (0.11), 0.82, 0.59–0.94**	**< 0.001**
Total follow-up length (months)	71.5 (33.3), 55, 36–122	70.7 (33.9), 63, 36–122	73.3 (31.9), 45.5, 36–122	0.18
From baseline to first Boolean remission (months)	8.1 (13.2), 4, 1–111	**2.7 (1.6), 2, 1**–**6**	**18.7 (8.1), 14, 7**–**111**	**< 0.001**
From first Boolean remission to last observation (months)	79.4 (38.6), 55, 4–121	**84.6 (42.3), 63, 30**–**121**	**71.3 (35.8), 46, 4**–**115**	**< 0.001**
Total consult times	48.7 (38.7), 40, 10–185	**46.2 (37.8), 39, 10**–**180**	**54.5 (40.2), 45, 10**–**185**	**< 0.05**

G ≤ 6, a patient group who achieved Boolean remission within 6 months from the baseline; G > 6, a patient group who achieved Boolean remission longer than 6 months from the baseline; *ACPA* anti-cyclic citrullinated polypeptide antibodies, *RF* rheumatoid factor, *MTX* methotrexate, *b/tsDMARD* biologic/targeted disease-modifying anti-rheumatic drug, *GCS* glucocorticoid steroid, *TJC* tenderness joint count, *SJC* swollen joint count, *PGA* patient's global assessment, *EGA* evaluator's global assessment, *CRP* C-reactive protein, *DAS28* 28-joints disease activity score, *CDAI* clinical disease activity index, *SDAI* simplified disease activity index, *HAQ-DI* Health Assessment Questionnaire Disability Index, *PS-VAS* pain score using visual analog scale, *SHS* Sharp/van der Heijde Score, *QOLS* quality of life score.

Units: age, year old; disease duration, years; CRP, mg/L; PS-VAS, millimeter. The other parameters' units are numerical values.

Except Female, ACPA positive, and RF positive, mean value (standard deviation), median, and range separated with comma are shown. In Female, ACPA positive, and RF positive, real numbers and their ratios in the parentheses are shown.

In Female, ACPA positive, and RF positive, *p*-values were calculated using chi square test, while in the other parameters, Mann–Whitney U-test for the statistical comparison between the G ≤ 6 and the G > 6 was used.

Statistically significant columns are shown in bold style.

Among the study parameters, disease duration, HAQ-DI score, PS-VAS, SHS, and QOLS were significantly correlated with the time length by univariate linear regression analysis. In addition, PS-VAS, SHS, and QOLS were significantly correlated with the time length by multivariate linear regression analysis (Table 2). In summary, the greater PS-VAS and SHS at baseline, the longer time length could be predicted, and the less QOLS at baseline.

**Table 2:** Relationship between TL and parameters at the baseline.

Parameters	Linear regression analysis				Binary logistic regression analysis in regard to the cut-off index of TL
	Univariate model		Multivariate model		
	Coefficients (95% CI)	*p*-value	Coefficients (95% CI)	*p*-value	Odds ratio
Age	0.037 (− 0.053 to 0.120)	0.45			
ACPA	0.000 (− 0.002 to 0.003)	0.80			
RF	0.001 (− 0.004 to 0.006)	0.61			
Disease duration	**0.181 (0.026** to **0.336)**	**< 0.05**	0.068 (− 0.229 to 0.365)	0.65	0.967
TJC	0.190 (− 0.218 to 0.598)	0.35			
SJC	− 0.007 (− 0.265 to 0.251)	0.96			
PGA	0.397 (− 0.041 to 0.835)	0.076			
EGA	0.308 (− 0.288 to 0.903)	0.31			
CRP	− 0.079 (− 0.531 to 0.372)	0.73			
DAS28	0.567 (− 0.475 to 1.609)	0.29			
CDAI	0.054 (− 0.062 to 0.171)	0.36			
SDAI	0.032 (− 0.068 to 0.133)	0.53			
HAQ-DI	**2.696 (0.596** to **4.796)**	**< 0.05**	− 2.208 (− 5.504 to 1.351)	0.23	0.999
PS-VAS	**0.064 (0.023** to **0.104)**	**< 0.01**	**0.097 (0.034** to **0.159)**	**< 0.01**	**0.988**
SHS	**0.052 (0.031** to **0.072)**	**< 0.001**	**0.060 (0.030** to **0.091)**	**< 0.001**	**0.994**
QOLS	− **19.792 (**− **31.55** to − **8.03)**	**< 0.01**	− **25.994 (**− **42.049** to − **9.938)**	**< 0.001**	**24.789**

*TL* time length from the baseline to the first Boolean remission, *ACPA* anti-cyclic citrullinated polypeptide antibodies, *RF* rheumatoid factor, *TJC* tenderness joint count, *SJC* swollen joint count, *PGA* patient's global assessment, *EGA* evaluator's global assessment, *CRP* C-reactive protein, *DAS28* 28-joints disease activity score, *CDAI* clinical disease activity index, *SDAI* simplified disease activity index, *HAQ-DI* Health Assessment Questionnaire Disability Index, *PS-VAS* pain score using visual analog scale, *SHS* Sharp/van der Heijde Score, *QOLS* quality of life score.

Statistical analyses were performed using univariate linear regression analysis as the time span was defined as dependent factor and the parameters were defined as independent factors, and then multivariate linear regression analysis was performed with the parameters that demonstrated significant correlation with the univariate model.

Statistically significant columns are shown in bold style.

### Preliminary regression analysis and ROC study for determining appropriate interval from baseline to first Boolean remission

**Figure 1:**
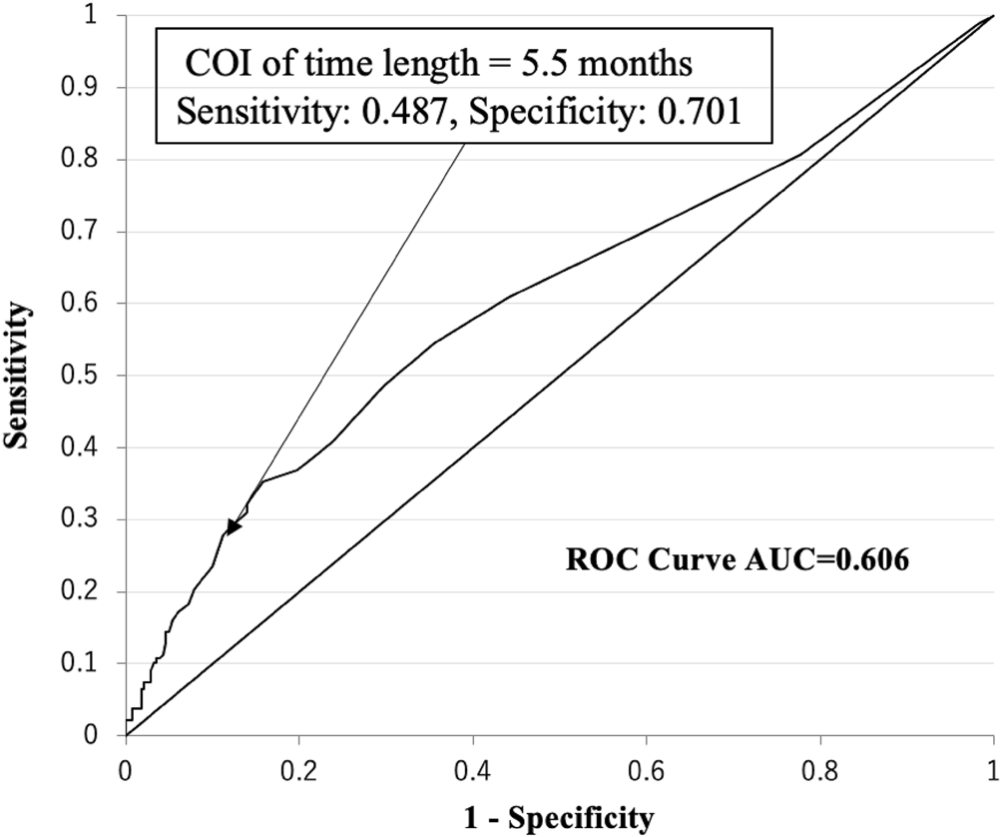
ROC curve of time length for attaining SDAI remission equivalent (SDAI ≤ 3.3) as a mean value after acquisition first Boolean remission of the patients analyzed in the study. Area under the curve was 0.606.

The time from baseline to first Boolean remission and the mean SDAI score after Boolean remission were significantly correlated (p < 0.001). In the Receiver Operating Characteristic Curve (ROC) study, 5.5 was shown as the cut-off index (COI) and the area under the curve was 0.606, with 0.487 and 0.701 of sensitivity and specificity, respectively (p < 0.001) (Fig. 1).

### Regression analysis at the first Boolean remission

Parameters such as HAQ-DI score, PS-VAS, SHS, and QOLS significantly correlated with the time length at the first Boolean remission, and SDAI, HAQ-DI score, PS-VAS, SHS, and QOLS after the Boolean remission correlated significantly with the time length. Thus, the time length correlated with all of these variants at the first remission and thereafter except for the SDAI score at the remission (Table 3).

**Table 3:** Correlation between the TL and clinical parameters at the First Boolean remission and between the TL and mean value of the parameters thereafter.

Parameters	At the first Boolean remission		After the first Boolean remission	
	Coefficients (95% CI)	*p*-value	Coefficients (95% CI)	*p*-value
SDAI	0.001 (− 0.005 to 0.008)	0.76	**0.041 (0.023** to **0.059)**	**< 0.001**
HAQ-DI	**0.009 (0.005** to **0.013)**	**< 0.001**	**0.009 (0.006** to **0.013)**	**< 0.001**
PS-VAS	**0.204 (0.061** to **0.348)**	**< 0.01**	**0.207 (0.100** to **0.317)**	**< 0.001**
SHS	**1.053 (0.670** to **1.436)**	**< 0.001**	**1.583 (1.023** to **2.144)**	**< 0.001**
QOLS	− **0.002 (**− **0.003** to − **0.001)**	**< 0.001**	− **0.002 (**− **0.003** to − **0.001)**	**< 0.001**

*TL* time length until the first Boolean remission, *SDAI* simplified disease activity index, *HAQ-DI* Health Assessment Questionnaire Disability Index, *PS-VAS* pain score using visual analog scale, *SHS* Sharp/van der Heijde Score, *QOLS* quality of life score.

Statistical analyses were performed using univariate linear regression analysis as the time span was defined as independent factor and the parameters were defined as dependent factors.

Statistically significant columns are shown in bold style.

The odds ratios for PS-VAS, SHS, and QOLS at baseline in regard to the COI of the time length were 0.988, 0.994, and 24.789, respectively, using binary regression analysis (Table 2).

### Comparison analysis

The comparison between the G ≤ 6 and the G > 6 groups revealed that the disease duration, HAQ-DI score, PS-VAS, and SHS at baseline in the G > 6 were significantly higher than that in the G ≤ 6 group, and QOLS in the G ≤ 6 group was significantly higher than that in the G > 6 group at baseline (Table 1). Similarly, the HAQ-DI score, SHS, and PS-VAS at the first Boolean remission in the G > 6 group were significantly higher than that in the G ≤ 6 group, whereas QOLS in the G ≤ 6 group demonstrated no significant difference compared with that in the G > 6 group. In summarize, the G > 6 group had different characteristics at baseline from the G ≤ 6 group had such as longer disease history, higher joint deformity, inferior pain, ALD, and QOL profile, yet no difference in disease activity between the two groups was shown. For treatment detail, mean MTX dosage and b/tsDMARD administration rate in the G > 6 group were significantly higher than those in the G ≤ 6 group at the first Boolean remission, despite there being no significant difference between the two groups at baseline. The other parameters showed no significant differences between the two groups (Table 4).

**Table 4:** Comparison of parameters at the first Boolean remission and mean values of parameter thereafter between the G ≤ 6 and the G > 6.

	At the first Boolean remission			After the first Boolean remission to last observation		
	G ≤ 6	G > 6	*p*-value	G ≤ 6	G > 6	*p*-value
MTX administration rate	70.3%	66.9%	0.47	77.5%	70.4%	0.10
Mean MTX dosage	**7.9**	**8.8**	**< 0.05**	8.2	8.3	0.84
GCS administration rate	29.4%	27.5%	0.67	29.6%	43.7%	0.24
Mean GCS dosage	3.2	3.3	0.86	3.3	3.9	0.30
b/tsDMARD administration rate	**13.3%**	**27.5%**	**< 0.001**	19.3%	20.4%	0.78
SDAI	1.03, 1.04	1.10, 1.00	0.49	**2.91, 2.40**	**4.05, 3.03**	**< 0.001**
SDAI remission rate	96.5%	96.5%	0.98	**72.2%**	**58.0%**	**< 0.001**
Boolean remission rate	100%	100%	1.00	**62.0%**	**43.4%**	**< 0.001**
HAQ-DI	**0.370, 0.551**	**0.552, 0.600**	**< 0.01**	**0.364, 0.510**	**0.567, 0.599**	**< 0.001**
PS-VAS	**13.0, 18.4**	**20.7, 24.5**	**< 0.001**	**17.7, 14.9**	**24.1, 17.7**	**< 0.001**
SHS	**27.3, 49.8**	**70.7, 79.5**	**< 0.001**	**26.5, 48.6**	**69.0, 77.8**	**< 0.001**
QOLS	0.845, 0.139	0.791, 0.156	0.79	**0.828, 0.108**	**0.765, 0.128**	**< 0.001**

G ≤ 6, a patient group who achieved Boolean remission within 6 months from the baseline; G > 6, a patient group who achieved Boolean remission longer than 6 months from the baseline; *MTX* methotrexate, *GCS* glucocorticoid steroid, *b/tsDMARD* biologic or targeted synthetic disease-modifying anti-rheumatic drug, *SDAI *simplified disease activity index, *HAQ-DI* Health Assessment Questionnaire Disability Index, *PS-VAS* pain score using visual analog scale, *SHS* Sharp/van der Heijde Score, *QOLS* quality of life score.

Units: time span, months; MTX dosage, mg per week; mean GCS dosage, mg per day; PS-VAS, millimeter. The other parameters' units are numerical values.

In time span at the first Boolean remission columns, the time spans from the baseline to the first Boolean remission for each group are expressed.

In time span after the first Boolean remission columns, the mean time spans from the first Boolean remission to the last observation for each group are expressed.

In time span, SDAI, HAQ-DI, PS-VAS, and QOLS, mean values and standard deviations separated with comma are shown.

In mean MTX dosage and mean GCS dosage, mean dosages in patients who was administered with each drug were shown.

*p*-values in time span, mean MTX dosage, mean GCS dosage, SDAI, HAQ-DI, PS-VAS, SHS, and QOLS, are calculated using Mann–Whitney U-test. The other statistics are calculated using chi square test.

Statistically significant columns are shown in bold style.

The mean value of the SDAI score after the first Boolean remission to the last observation demonstrated a significant increase from the first Boolean remission in both groups and in the G > 6 group was significantly higher than that in the G ≤ 6 group. Similarly, the SDAI score, the HAQ-DI score, PS-VAS, and SHS after the first Boolean remission to the last observation in the G > 6 group were also significantly higher than those in the G ≤ 6 group, and the mean value of the QOLS in the G ≤ 6 group was significantly higher than that in the G > 6 group. The Boolean remission rate and SDAI remission rate after the first Boolean remission to the last observation were significantly higher in the G ≤ 6 group than those in the G > 6 group (Table 4). The change of the SDAI score from the first Boolean remission to after the remission was significantly lower in the G ≤ 6 group than that in the G > 6 group, whereas the changes in the HAQ-DI score, PS-VAS, SHS, and QOLS demonstrated no significant differences between the two groups (Table 5).

**Table 5:** Absolute value and change of parameters for each group at each moment.

		At baseline	Change at interval	At Boolean remission	Change at interval	After Boolean remission	p-value
SDAI	G ≤ 6	13.3 (12.9)	− 11.0 (12.7)***	1.03 (1.04)	1.88 (2.40)***	2.91 (2.40)	0.68, 0.49, ***
	G > 6	13.3 (9.8)	− 11.5 (9.7)***	1.10 (1.00)	2.96 (2.98)***	4.05 (3.03)	
HAQ-DI	G ≤ 6	0.418 (0.564)	− 0.045 (0.246)***	0.370 (0.551)	− 0.006 (0.232)***	0.364 (0.510)	**, ***, ***
	G > 6	0.578 (0.607)	− 0.029 (0.315)***	0.552 (0.600)	0.015 (0.254)	0.567 (0.599)	
PS-VAS	G ≤ 6	30.3 (28.9)	− 17.0 (28.0)***	13.0 (18.4)	4.7 (15.7)***	17.7 (14.9)	***, ***, ***
	G > 6	40.5 (29.6)	− 19.9 (31.9)***	20.7 (24.5)	3.4 (19.0)***	24.1 (17.7)	
SHS	G ≤ 6	28.3 (51.3)	0.0 (0.5)***	27.3 (49.8)	− 0.2 (1.5)***	26.5 (48.6)	***, ***, ***
	G > 6	69.1 (78.9)	− 0.4 (7.5)	70.7 (79.4)	− 0.2 (1.7)*	69.0 (77.8)	
QOLS	G ≤ 6	0.847 (0.131)	0.073 (0.134)***	0.845 (0.139)	− 0.017 (0.075)***	0.828 (0.108)	***, 0.79, ***
	G > 6	0.805 (0.113)	0.018 (0.130)***	0.791 (0.139)	− 0.026 (0.084)	0.765 (0.128)	

Standard deviations in parentheses are shown.

In change at interval rows, *, **, and *** represented statistical significance of p < 0.05, p < 0.01, and p < 0.001 respectively, for which were evaluated time to time change using ANOVA with repeated measures.

In p-value rows, *, **, and *** represented statistical significance of p < 0.05, p < 0.01, and p < 0.001 respectively, for which were evaluated differences between G ≤ 6 and G > 6 group for each moment at baseline, Boolean remission, and after Boolean remission, respectively.

*SDAI* simplified disease activity index, *HAQ-DI* Health Assessment Questionnaire Disability Index, *PS-VAS* pain score with visual analog scale, *SHS* Sharp/van der Heijde score, *QOLS* quality of life score.

**Figure 2:**
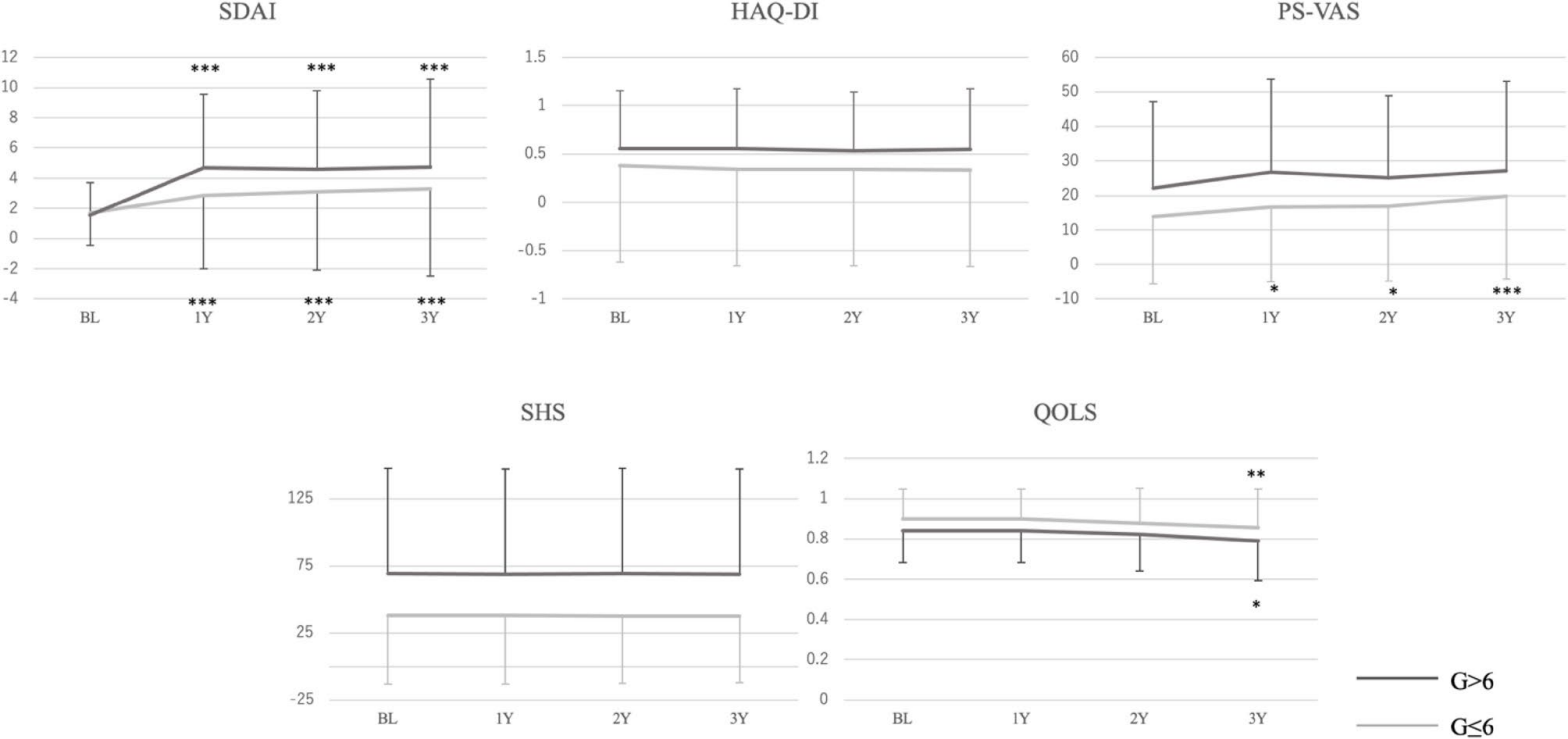
Time courses of the parameters from the first Boolean remission to 3 years after the remission comparing in the G ≤ 6 and in the G > 6. Error bars that show standard deviation in each group were shown at each moment. Except for the SDAI score at the first Boolean remission, mean values of all parameters at any moment were significantly lower in the G ≤ 6 group than those in the G > 6 group (p < 0.001), and the QOLS was significantly higher at any moment in the G ≤ 6 group the those in the G > 6 group at any moment (p < 0.01). Change of mean SDAI score was significantly lower in the G ≤ 6 than in the G > 6 (*p* < 0.001), and change of the other parameters such as HAQ-DI, PS-VAS, SHS, and QOLS demonstrated no significant difference between the two groups. Statical significances of time change at each moment after the first Boolean remission (BL) for each parameter in the each group compared to the values at the BL were symbolized in the figure (*p < 0.5; **p < 0.01; ***p < 0.001).

Except for SDAI at the first Boolean remission, all parameters such as SDAI, HAQ-DI, PS-VAS, and SHS at any moment from one to three years after in the G ≤ 6 group were significantly lower than those in the G > 6 group (p < 0.001), and QOLS in the G ≤ 6 group was significantly higher than that in the G > 6 group (p < 0.01). The change value of the SDAI score in the G ≤ 6 group was significantly lower than that in the G > 6 group at any moment from one to three years after the first Boolean remission (p < 0.001). The SDAI scores at one to three year after compared to that at the first Boolean remission were significantly higher in both groups (p < 0.001), whereas the PS-VAS after the first Boolean remission was significantly higher than that at the remission, and the p-values were < 0.05, < 0.05, and < 0.001 at one, two, and three years after, respectively. The QOLS three years after compared to that at the first remission was significantly lower in both groups, and the p-value was < 0.01 and < 0.05 in the G ≤ 6 group and G > 6 group, respectively (Fig. 2).

### Relationship between Boolean remission rate and the time length, and other parameters

**Figure 3:**
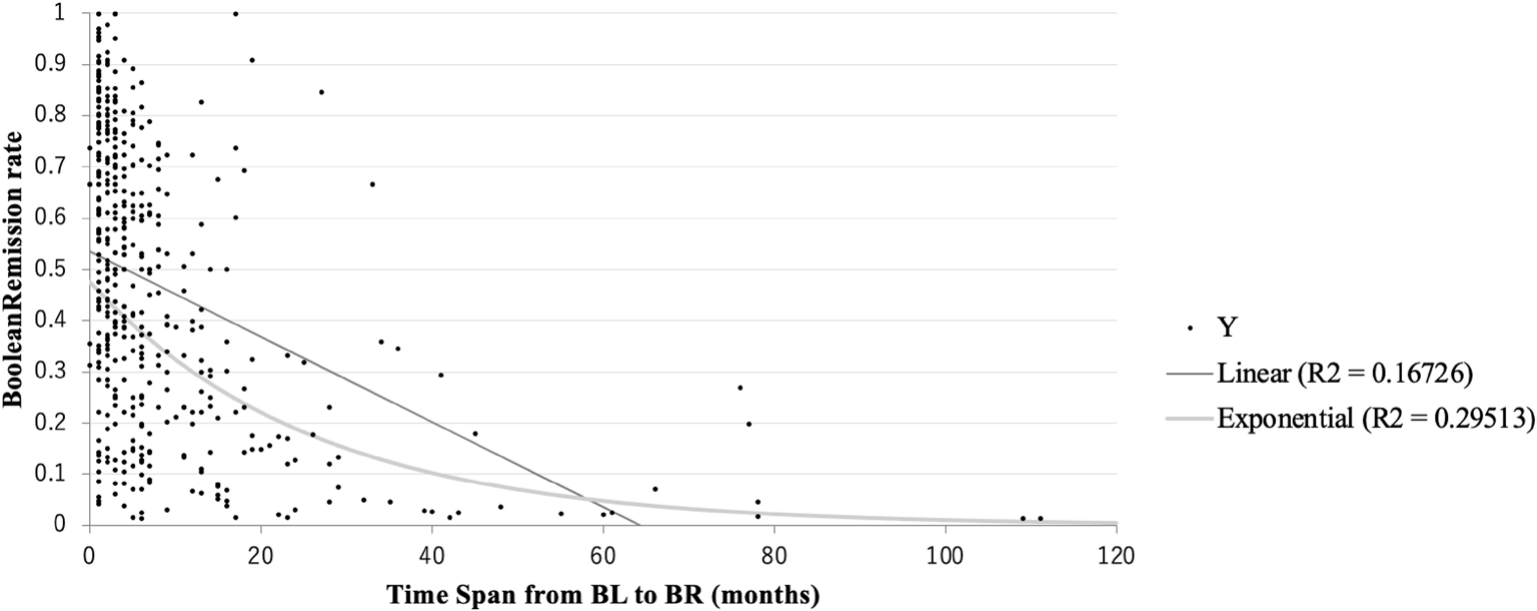
Relationship between total Boolean remission rate (Y-axis) and time length to the first Boolean remission (X-axis). Approximate equations were linear and exponential equations, and correlation coefficient was 0.4090 and 0.5467, respectively.

A scatter plot of the relationship between the overall Boolean remission rate and the length of time in each case is shown in Fig. 3. At a glance, the longer the interval from baseline to the first Boolean remission, the lower the Boolean remission rate. The most highly correlated approximation was the exponential equation, with a correlation coefficient of 0.5433, while the linear equation had a correlation coefficient of 0.4090, in which the two parameters were significantly highly correlated (p < 0.001).

**Figure 4:**
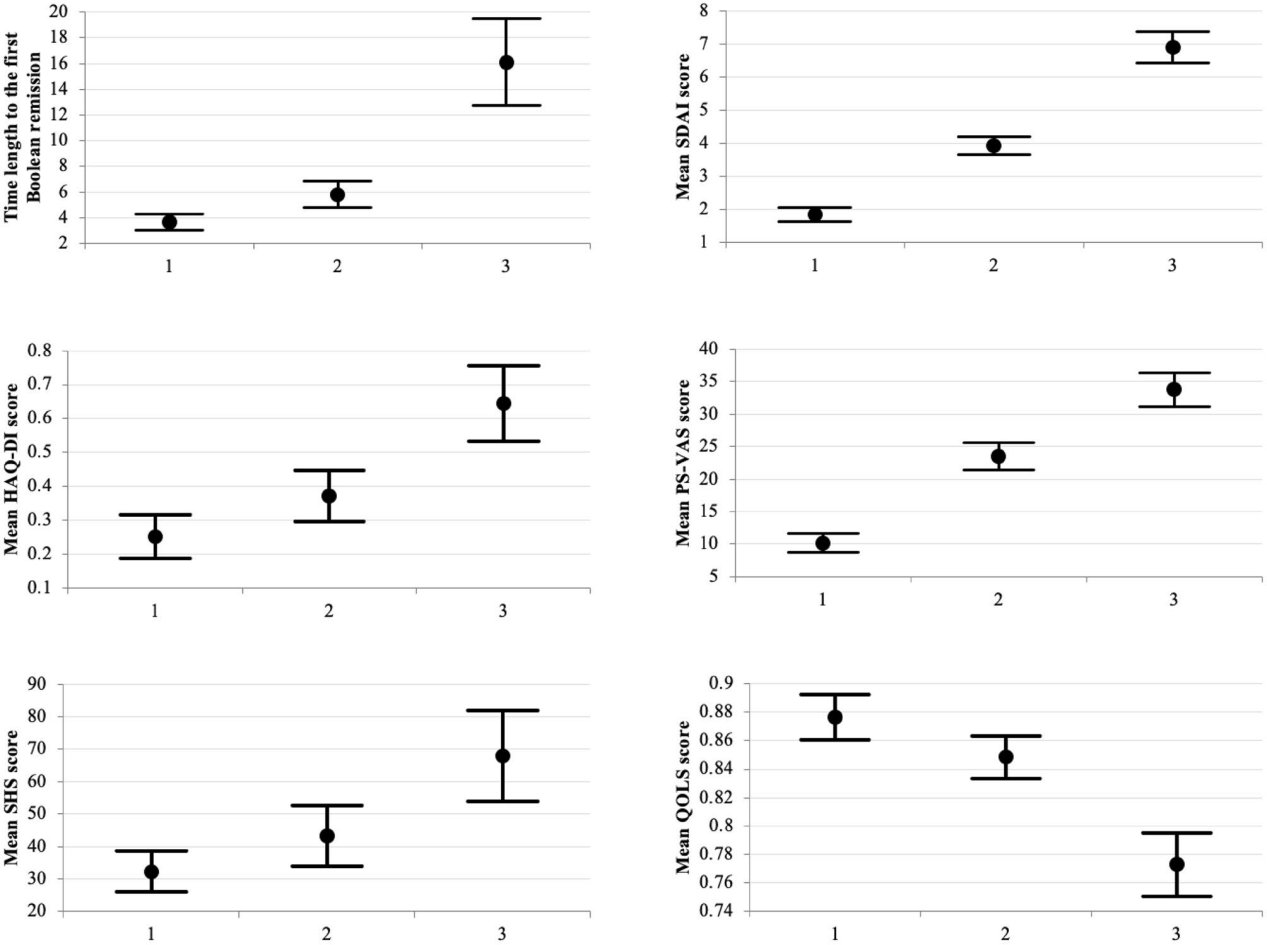
Comparison among Boolean remission rate groups. (1) a patient group whose Boolean remission rate was 60% or more; (2) a patient group whose Boolean remission rate was less than 60% and 30% or more; (3) a patient group whose Boolean remission rate was less than 30%. Error bars represent 95% confidence interval (95% CI). Every parameter in the group 3 demonstrated significantly worse results than those in the other two groups.

The mean SDAI score, PS-VAS, and SHS demonstrated significantly greater values in group 3 (a patient group whose Boolean remission rate was less than 30%) than in group 2 (a patient group whose Boolean remission rate was less than 60% and 30% or more) and group 1 (a patient group whose Boolean remission rate was 60% or more), and demonstrated significantly greater values in the group 2 than in the group 1, whereas the mean time length and HAQ-DI score demonstrated significantly greater in the group 3 than in the group 2 and group 1 and the mean value of the QOLS demonstrated significant less in the group 3 than in the group 2 and group 1 (Fig. 4 and Table 6).

**Table 6:** Comparison between each pair of the groups.

	Group1	Group2	Group3	p-value		
				1 vs 2	1 vs 3	2 vs 3
TL (months)	3.7 (3.0–4.3)	5.8 (4.8–6.9)	16.1 (12.7–19.5)	0.32	< 0.001	< 0.001
SDAI	1.84 (1.62–2.06)	3.92 (3.65–4.20)	6.91 (6.45–7.38)	< 0.001	< 0.001	< 0.001
HAQ-DI	0.251 (0.187–0.315)	0.372 (0.296–0.447)	0.644 (0.532–0.755)	0.13	< 0.001	< 0.001
PS-VAS	10.2 (8.8–11.6)	23.5 (21.3–25.7)	33.8 (31.2–36.3)	< 0.001	< 0.001	< 0.001
SHS	32.3 (25.9–38.7)	43.3 (34.1–52.5)	68.0 (54.1–81.9)	< 0.001	< 0.001	< 0.001
QOLS	0.876 (0.860–0.892)	0.848 (0.833–0.863)	0.773 (0.750–0.795)	< 0.001	< 0.001	< 0.001

Statistical procedures: Student T-test.

In columns, mean value and 95% confidence interval in parentheses are shown.

Statistical procedure: Student T-test.

Columns show initial mean, 95% confidence interval in parentheses.

All parameters are calculated as means.

*TL* time from baseline to first Boolean remission, *SDAI* simplified disease activity index score, *HAQ-DI* Health Assessment Questionnaire Disability Index score, *PS-VAS* pain score using visual analog scale, *SHS* Sharp/van der Heijde score, *QOLS* quality of life score calculated from EuroQol 5th-dimension score.

## Discussion

RA is a chronic inflammatory disease that involves the joint structure, and this makes ADL difficult. Therefore, controlling inflammation in the early stage is recommended^2–5, 28^, because early drug intervention prevents joint destruction due to persistent disease activity and the resulting damage to ADL by controlling disease activity with close monitoring of the objective and subjective disease activity^29^. Although progress in rheumatology has been remarkable, improvement in RA treatment is still the most important issue^30^.

Inflammation control is also present in the overarching principle, which is first mentioned in the EULAR recommendation for managing RA^2–5^. This leads to the aim of the goal of clinical remission within 3–6 months^3–5^. All patients recruited in this study had been under the T2T strategy, which includes monitoring the patient’s disease activity, ADL, and comprehensive condition, and considering treatment protocol upon shared decision-making with the patient in order to fulfill clinical remission.

Indeed, obtaining clinical remission can prevent joint destruction and impairment in ADL, and many studies have reported many facts that progression is prevented by obtaining clinical remission, particularly in early RA^31–33^. The importance of monitoring and recording disease activity is much more thorough in the digital era^34^. Disease activity improvement with optimal discriminatory ability was suggested after 6 months in a clinical trial^35^. A study described the benefit of tight disease control that achieved DAS28-CRP remission significantly earlier compared with conventional treatment for patients with early RA^27^. Tight disease control, namely treatment under the T2T strategy, can achieve earlier clinical remission, and it might lead to a more stable clinical course. However, to the best of our knowledge, no study has evaluated the effect of time to remission on the subsequent clinical course in cases with clinical remission using the T2T treatment strategy.

The high Boolean remission achievement rate in the study was surprising. However, using the real-world data, based on the T2T strategy, out of 685 patients during the relatively long follow-up period of > 3 years, 465 (67.9%) showed Boolean remission once or more, it is realistic, because patients were picked up from various background. That is different from clinical trial study background. Therefore, it can be considered that such a high rate in both of G ≤ 6 and G > 6 groups are realistic given that the Boolean remission achievement rate after the first Boolean remission to the last observation was 62.0% and 43.4%, respectively. However, it is also a fact that some patients could not unfortunately achieve clinical remission, or Boolean remission for some reason such as the reason of patient's personal characteristics, or for some refractory disease status. These patients were excluded from the study.

The reason why 6 months is the critical cut-off index was as follows. Prior to this study, we preliminarily analyzed our data for determining a cut-off index of the time length for the best SDAI course thereafter. The results showed 5.5 months was the best length as a COI as shown in Fig. 1, so we determined 6 months as a critical COI in this study. The AUC of 0.6 for ROC is far from reliable. In fact, COI had a sensitivity of 48.7% and a specificity of 70.1%. However, the p-value was less than 0.1%, and there was a clear correlation between higher SDAI remission rates and shorter time to Boolean remission. As shown in Fig. 4, the time to remission was clearly shorter when the Boolean remission rate was higher. Not only that, the higher Boolean remission rate was clearly advantageous for ADL, pain, joint destruction, and QOL. Furthermore, the higher remission rate was also reported to have a critical impact on the prevention of fragility fractures in RA patients^36^.

As shown in Table 3, not only disease activity but also the shorter time to Boolean remission has a clear positive impact on ADL, joint destruction, and QOL after remission. Furthermore, as shown in Table 4, when divided by 6 months, all clinical indicators after achieving remission show that the group of 6 months or less is better than the group of 6 months or more. These results suggest that separating 6 months from COI leads to stable and comprehensive clinical outcomes and support the rationale for setting the goal of achieving clinical remission at 3–6 months, as demonstrated by T2T.

This study aimed to answer the clinical question of whether a shorter time span to achieve clinical remission would be necessary to make a successful outcome after remission, and how short it is. The primary endpoint was the disease activity after remission between the group of patients who achieved Boolean remission within 6 months after the diagnosis of RA and those who required > 6 months. These results showed that the group who achieved it within 6 months showed significantly better disease activity compared with the group that required > 6 months. The secondary endpoints of the HAQ-DI score, PS-VAS, SHS, and QOLS also showed significantly superior results. However, above all, these parameters were significantly superior in the group that achieved remission within 6 months even at the baseline, and these differences were maintained throughout the treatment.

In the study, the primary endpoint was set as the SDAI score, but not the DAS28 score. The reason for this was a difference in strictness. The remission criteria by means of SDAI is more stringent than those of DAS28-CRP, so as per previously published report quite different populations were recruited between the Boolean and the DAS28 remission, while similar populations were recruited between the Boolean and the SDAI remission^25, 37^.

QOL is also an important issue in treating RA because it directly correlates with work status^38^. Therefore, measuring QOL is important for monitoring disease status in RA treatment, as well as measuring physical function^39^. QOLS is calculated based on EQ-5D, which is a conversion formula applied to each disease in each country. QOLS is utilized in the study is used as a reference index when calculating cost–benefit for treating chronic low back pain^40^. To the best of our knowledge, this report is the first to be utilize QOLS to evaluate treatment outcomes in patients with RA, and it showed excellent reproducibility of QOL quantitative evaluation with convenient few questionnaire items and should be considered as a QOL index in patients with RA.

Disease duration and HAQ-DI as patient background factors were also significantly associated with the time length in univariate analysis, similar to Aletaha et al. study^33^, but PS-VAS, SHS, and QOLS were shown to be associated by multivariate analysis. These three parameters showed significant differences even at baseline, at the first Boolean remission, and after the first Boolean remission in comparison between the two groups, and the results strongly suggest that these factors correlated with the time length. However, these factors did not show any difference between the two groups regarding the change after the acquisition of Boolean remission, and the parameters that were affected by time length were SDAI remission rate, Boolean remission rate, and disease activity control after the achievement of Boolean remission. It is presumed that the significant difference in HAQ-DI and Boolean remission after the acquisition of at the first Boolean remission was because many cases with higher treatment resistance were included in the G > 6 group.

MTX dose and b/tsDMARD administration rate were significantly higher in the G > 6 group, despite these parameters demonstrated no significant difference between the two groups at baseline. This may be because the goal of Boolean remission resulted in the need for more intensive treatment compared with the G ≤ 6 group. However, the patient’s drug adherence was not considered in the study. There is a wide variability of drug adherence in patients, which strongly influences clinical results^41^. Previous treatment including b/tsDMARD administration at baseline did not influence on the time length. Like these, treatment initiation before disease activity gets high may have no influence on the time length because no disease activity difference at baseline was demonstrated between the two groups. The treatment protocol in the study was commonly designed under the T2T strategy, so every patient recruited in the study accepted shared decision-making and had been treated in targeted clinical remission. It seems to be clear that patient-related outcomes (PRO) such as PS-VAS and QOLS, are rather important for obtaining shorter time length. These parameters and the SHS score throughout treatment from baseline to after the first Boolean remission acquisition demonstrated a significant correlation with the time length. These results suggested that a patient who has good PROs from the baseline is well responsible for treatment when tight disease control is targeted.

Even acquisition of Boolean remission, sustaining the remission is obviously important. The only temporary achievement of Boolean remission is inferior to sustaining Boolean remission. The results of the relationship between the Boolean remission rate and other parameters showed that every parameters in  the lower remission rate group demonstrated more inferiority  than those in the other groups, while the lower remission rate group also demonstrated significantly longer time length than the two higher remission rate groups as shown in Fig. 4. These results suggested that the time length reflected more sensitivity in clinical outcomes including disease activity control and PRO.

Overall, the validity of aiming for clinical remission within 3–6 months under the T2T strategy has been shown to be effective. The strategy leads to the maintenance of disease activity as well as disease control. This in turn appeared to correlate with the maintenance of ADL and prevention of altered QOL. However, it is conceivable that achieving Boolean remission leads to maintenance. These prospects have not yet been proven in the study and require further investigation.

But the population in the study was a bit trickier. The non-SDAI indices HAQ-DI, PS-VAD, SHS, and QOLS were significantly better in the G ≤ 6 group than in the G > 6 group, and this trend persisted after the remission. The parameters improved until the acquisition of Boolean remission and progressively deteriorated after acquisition (Table 5). These parameters after the first remission were significantly correlated with the time length, as shown in Table 3, and parameters other than the SDAI score already showed the same trend at baseline. One confounding factor was mean disease duration at baseline because G > 6 was significantly longer than G ≤ 6. Duration of disease was significantly correlated with all parameters except QOLS (Table 7). This suggested that patients with a longer history obtained a Boolean remission but had a relatively worse clinical course than those with a shorter history.

**Table 7:** Correlation between parameters and disease duration at baseline.

Parameter	R	Coefficients (95% CI)	p-value
SDAI	0.141	− 0.216 (− 0.355 to − 0.076)	< 0.01
HAQ-DI	0.159	0.012 (0.005 to 0.019)	< 0.001
PS-VAS	0.115	− 0.428 (− 0.174 to − 0.087)	< 0.05
SHS	0.606	15.73 (6.48 to 15.42)	< 0.001
QOLS	0.101	− 0.002 (− 0.003 to 0.00004)	0.055

*95% CI* 95% confidence interval, *SDAI* simplified disease activity index, *HAQ-DI* Health Assessment Questionnaire Disability Index, *PS-VAS* pain score using visual analog scale, *SHS* Sharp/van der Heijde Score, *QOLS* quality of life score.

There are some limitations to the study. This was a single institutional study in which no ethnic and gender considerations were set and the influence of subjective comorbidities, such as depression, anxiety, fatigue, fibromyalgia, and other diseases were not considered^42^. However, this study addresses the effect of time length from the initiation of treatment to achieve Boolean remission for patients with RA.

In conclusion, we have investigated the impact of time length from baseline to achieve Boolean remission on disease activity control, ADL, and QOL maintenance with a cohort study. Results demonstrated that the shorter period to achieve remission, the tighter the disease activity is controlled, and the better ADL and QOL are maintained. Six months may be the key to guaranteeing a more stable clinical course after the acquisition of Boolean remission.

## Methods

### Treatment protocol

We have treated RA patients with RA since August 2010 under the T2T strategy in accordance with the EULAR recommendations in the institute where the only specialized clinic for rheumatic diseases in the community. All patients met the American College of Rheumatology/EULAR (ACR/EULAR) classification criteria^43^ at the initiation of RA treatment in the institute. The mean time length from the first visit to diagnosis was 1 week, and all RA patients were treated immediately after the diagnosis under the T2T treating strategy in the institute. We set a time of initiation of treatment in the institute as a baseline.

Patients were consulted and treated every 2–3 months intervals since the diagnosis of RA. They were monitored for their tenderness joint count (TJC), swollen joint count (SJC), patient’s global assessment (PGA), evaluator’s global assessment (EGA), C-reactive protein (CRP), and disease activity indices, such as CDAI, SDAI, DAS28-CRP, and Boolean criteria at every visit to the institute. HAQ-DI score^44^, PS-VAS, and EuroQOL-5th dimension-5L (EQ-5D) were also monitored, and the QOLS calculated from EQ-5D^45^ was determined at every visit from the time of diagnosis. As an index of joint damage caused by RA, SHS^46^ was measured using X-ray pictures at the time of diagnosis and every other year, and within three months after the achievement of the first Boolean remission. SHS was confirmed by two physicians. The treatment protocol was considered every 3–6 months based on these clinical metrics and decided with a patient in sharing this information.

### Patient and parameters selection, and regression analysis at baseline

In patients with RA who had been treated in our institute under the T2T strategy, we enrolled the patients who had achieved Boolean remission once or more, which is defined as all of four parameters such as TJC, SJC, PGA, and CRP ≤ 1 and were consecutively followed up for > 3 years, in the observational study. The time length from the first visit to the first Boolean remission was calculated. The relationship between the time length and each of the background parameters at baseline such as sex, age, disease duration of RA, SHS at the first visit, ACPA, rheumatoid factor (RF), TJC, SJC, PGA, EGA, CRP, SDAI, HAQ-DI, PS-VAS, SHS, and QOLS were evaluated statistically using univariate linear regression analysis, and then multivariate linear regression analysis was performed to evaluate the relationship between the time length and the parameters that demonstrated significant correlation in the univariate model. All data were collected retrospectively from the medical record.

### Preliminary regression analysis and ROC study for determining appropriate interval from baseline to first Boolean remission

Before analyzing correlations, an association between time length from baseline to first Boolean remission and the mean SDAI score after the acquisition of Boolean remission was analyzed using linear regression analysis with a univariate model. After confirmation, the ROC study was conducted to determine the cut-off index (COI) for the length of time from baseline to first Boolean remission, demonstrating the most sensitivity and specificity values in SDAI remission rates as the mean value of SDAI after the first Boolean remission.

### Regression analysis at the first Boolean remission

The relationship between the time length and each of the mean values of the SDAI score, the HAQ-DI score, PS-VAS, SHS, and QOLS at the first Boolean remission and thereafter was then evaluated using univariate linear regression analysis considering each parameter as a dependent factor and the time length as an independent factor.

Furtherly, odds ratios of the parameters at the baseline, which demonstrated a significant correlation with the time length to achieve the first Boolean remission, were evaluated using binary logistic regression analysis compared with the time length to attain Boolean remission separated in 6 months.

### Comparison analysis

Patients were subsequently divided into the G ≤ 6 and G > 6 groups based on the time length for the achievement of first Boolean remission within two groups: G ≤ 6, a patient group who attained Boolean remission within 6 months from the first visit; G > 6, a patient group who attained Boolean remission more than 6 months from the first visit. The two groups were compared with regard to the SDAI score, the HAQ-DI score, PS-VAS, SHS, and QOLS at the first visit and at the time of first Boolean remission, and the values of these parameters at 1–3 years and the mean values of these parameters after the first Boolean remission were assessed using the Mann–Whitney U test. Repeated measures of ANOVA were used for statistical procedures to evaluate the change of these parameters between the moments. Methotrexate (MTX), biologic/targeted disease-modifying anti-rheumatic drug (b/tsDMARD), and glucocorticoid steroid (GCS) administration rate at the first visit were also compared between the two groups using Mann–Whitney U-test. Moreover, changes in these parameters from the first Boolean remission to thereafter between the two groups were also compared using the Mann–Whitney U test. Rates of treatment with mean doses of b/tsDMARD, MTX, and GCS administration rate and mean dose of administration at the first Boolean remission and thereafter between the two groups were also compared using the Mann–Whitney U and chi-square tests. The mean Boolean remission rate after the first remission, and SDAI remission rate at the first Boolean remission and thereafter were also compared between the two groups using the Mann–Whitney U test. The primary endpoint was the mean value of the SDAI score after the first Boolean remission to the last observation, and secondary endpoints included the mean values of the HAQ-DI score, PS-VAS, SHS, and QOLS after the first Boolean remission.

### Relationship between Boolean remission rate and the time length, and other parameters

A relationship between the total Boolean remission rate and the time length for each case was plotted with scattered graphs and evaluated with an approximate curve equation, that showed maximum correlation coefficients. Based on the equation, patients were classified into three groups according to Boolean remission rate; 1, a patient group whose Boolean remission rate was 60% or more; 2, a patient group whose Boolean remission rate was less than 60% and 30% or more; 3, a patient group whose Boolean remission rate was less than 30%. Mean values of the time length, SDAI, HAQ-DI, PS-VAS, SHS, and QOS of these three groups were compared using T-test.

The study protocol was approved by the ethics committee of the study institution (approval number: Y-2020-RA-2). All methods were performed in accordance with the Declaration of Helsinki and other relevant guidelines and regulations.

### Software used in the statistical procedures

All the statistical procedures were performed using StatPlus:mac^®^ (AnalystSoft, Inc., Walnut, CA, USA), and significance level was set within 5%.

### Ethics and consent

The study protocols and patient consent requirements were approved by Yoshii Hospital Ethics Committee (approval number: Y-2020-RA-2). The subjects and their families were informed that the personal information obtained in this study was anonymous and would only be used for analysis. Informed consent was obtained from all participants enrolled in the study and all subjects and their families provided signed consent.

## Data Availability

The datasets used and/or analyzed during the current study are available from the corresponding author on reasonable request.

## Abbreviations

RA: Rheumatoid arthritis
SDAI: Simplified disease activity index
CDAI: Clinical disease activity index
DAS28-CRP: 28-Joint disease activity score using C-reactive protein
ADL: Daily activities in life
QOL: Quality of life
EULAR: The European League Against Rheumatism
T2T: Treat to target
TJC: Tenderness joint count
SJC: Swollen joint count
PGA: Patient’s global assessment
EGA: Evaluator’s global assessment
CRP: C-reactive protein
HAQ-DI: Health Assessment Questionnaire Disability Index
PS-VAS: Pain score using visual analog scale
EQ-5D: EuroQOL-5th dimension-5L
QOLS: Quality of life score
SHS: Sharp/van der Heijde score
ACPA: Anti-cyclic citrullinated peptide antibodies
RF: Rheumatoid factor
b/tsDMARD: Biologic/targeted synthetic disease-modifying anti-rheumatic drug
MTX: Methotrexate
GCS: Glucocorticoid steroid
PRO: Patient related outcomes
